# Shared evidence, different recommendations: a comparative analysis of Qdenga (TAK-003) dengue vaccine guidance for travellers across countries

**DOI:** 10.1093/jtm/taag065

**Published:** 2026-07-22

**Authors:** Sarah L McGuinness, Dipti Patel, Helena H Askling, Camilla Rothe, Steven Schofield, Robert Steffen, Leo G Visser, Lin H Chen

**Affiliations:** School of Public Health and Preventive Medicine, Monash University, 553 St Kilda Road, Melbourne, VIC, 3004 Australia; Department of Infectious Diseases, The Alfred and School of Translational Medicine, Monash University, 55 Commercial Road, Melbourne, VIC 3004, Australia; National Travel Health Network and Centre (NaTHNaC), University College London Hospitals, 250 Euston Road, London NW1 2PG, UK; Department of Medicine, Division of Infectious Diseases, Karolinska Institutet, K2 Medicin, Solna 171 77 Stockholm, Sweden; Academic Specialist Centre, Stockholm Health Care Services, Solnavägen 1E, 113 65 Stockholm, Sweden; Institute of Infectious Diseases and Tropical Medicine, LMU University Hospital, 80802 Munich, Bavaria, Germany; Department of National Defence, Government of Canada, 101 Colonel By Drive, Ottawa, ON K1A 0K2, Canada; Epidemiology, Biostatistics and Prevention Institute, Department of Public and Global Health, WHO Collaborating Centre for Travelers’ Health, University of Zurich, Hirschengraben 84, CH-8001, Zurich, Switzerland; Division of Epidemiology, Human Genetics & Environmental Sciences, School of Public Health, University of Texas, 7000 Fannin, Houston, TX 77030, USA; Dutch Coordination Centre for Travellers Health Advice, 1018 WT Amsterdam, The Netherlands; Leiden University Centre for Infectious Diseases, Department of Infectious Diseases, Leiden University Medical Centre, 2333 Leiden, The Netherlands; Department of Medicine, Mount Auburn Hospital, 330 Mount Auburn Street, Cambridge, MA 02138, USA; Faculty of Medicine, Harvard Medical School, 25 Shattuck Street, Boston, MA 02115, USA

**Keywords:** guidelines, decision-making, risk assessment, travel vaccination, dengue vaccines, serology, NITAG

## Abstract

**Introduction:**

Dengue is an increasingly important cause of illness in travellers, and Qdenga (TAK-003) is the first dengue vaccine incorporated into guidance for travellers in multiple countries. However, clinicians and travellers may encounter differing recommendations across jurisdictions. This study compared guidance on the use of Qdenga in travellers across countries.

**Methods:**

We conducted a comparative analysis of national guidance on Qdenga use for travellers across countries, using survey data and structured review of publicly available recommendations. Guidance was synthesized according to dengue immune status and mapped across recurring decision domains.

**Results:**

Survey responses were obtained from 18 advisory bodies across 17 countries between March and April 2026, including 12 non-endemic countries where Qdenga was licensed and guidance published. Guidance was generally more restrictive than regulatory authorization. While there was broad agreement in recommending vaccination for travellers with laboratory-confirmed prior dengue infection, recommendations for those without documented infection varied widely. All advisory bodies considered destination-specific dengue risk but differed in whether and how they used travel duration, repeated or cumulative exposure, traveller characteristics and host susceptibility factors to determine vaccine eligibility. Some recommendations restricted vaccination to travellers with prior dengue infection, whereas others extended eligibility to selected travellers without documented prior infection or recommended vaccination based on exposure risk, irrespective of serostatus. Approaches also differed in assessment of prior dengue infection and whether completion of the two-dose series before travel was required.

**Conclusions:**

Variation in Qdenga guidance reflects differences in how advisory bodies interpret shared evidence and manage uncertainty arising from data gaps and differences between trial populations and travellers. Additional data on vaccine effectiveness and clinical outcomes among seronegative travellers and older adults may help reduce uncertainty and support greater alignment of future recommendations. Regardless, transparency and shared clinical decision-making remain essential when discussing dengue vaccination with travellers.

## Introduction

The global burden of dengue has risen dramatically since 1990, reaching a record of more than 14 million reported cases in 2024, including 11 201 deaths.^1^ This rise has been linked to multiple factors including the changing climate, urbanization and increased global mobility.^2^ In parallel, dengue has become a leading cause of febrile illness among international travellers.^3–5^ As a result, dengue prevention is now a key component of pre-travel consultations, although, until recently, it relied almost entirely on measures to reduce exposure to mosquito bites.^6^

Dengue vaccine development has aimed to provide protection against all four dengue virus serotypes while ensuring safe and effective use across all populations, including individuals without prior dengue infection.^7^ This has been challenging, particularly given the risk of immune-mediated enhancement of disease following secondary infection.^8^^,^^9^ Three live-attenuated recombinant tetravalent dengue vaccines have now been licensed: CYD-TDV (Dengvaxia®, Sanofi), TAK-003 (Qdenga®, Takeda) and TV003 (Butantan-DV, Instituto Butantan).^2^ Post-licensure experience with CYD-TDV demonstrated an increased risk of severe dengue among seronegative vaccine recipients following natural infection, leading to restrictions on its use to individuals with confirmed prior dengue infection.^10^ Sanofi has since announced discontinuation of CYD-TDV manufacture and supply for commercial reasons.^11^ TV003 has only been authorized in Brazil for use in endemic populations, but distribution is temporarily suspended.^12^ Qdenga is the only dengue vaccine incorporated into guidance for international travellers across multiple countries.^2^^,^^13^ In countries where it is licensed, it is approved for use in individuals aged 4 years and older without restrictions based on prior dengue infection. The vaccine is based on an attenuated DENV-2 backbone into which proteins from DENV-1, DENV-3 and DENV-4 are incorporated to generate immune responses against all four dengue virus serotypes.^11^ Qdenga is administered as a two-dose series, 3 months apart. It is authorized in more than 40 countries including in Europe and Australia but not in the USA, Canada or Japan.^7^

In contrast to populations living in dengue-endemic settings, most international travellers are immunologically naïve to dengue.^14^ Qdenga vaccine performance varies according to prior dengue infection status, making serostatus an important consideration in forming recommendations.^15^ A key challenge in developing guidance for travellers is that evidence for Qdenga efficacy comes primarily from studies in children and adolescents living in dengue-endemic areas who are often seropositive, whereas travellers span all age groups and are typically seronegative.^14^^,^^16^^,^^17^ Travel-related dengue exposure is also more heterogenous than in endemic populations. Although many travellers have relatively brief exposure, repeated or prolonged travel—particularly among frequent, long-term or occupational travellers—increases the likelihood of infection and clinical disease.^15^^,^^18^

Among non-endemic countries where Qdenga is authorized and/or guidance exists, national approaches to clinical recommendations differ with respect to eligibility criteria, indications and implementation. In an increasingly connected and mobile world, clinicians may need to interpret and reconcile guidance from different jurisdictions, which may differ in their treatment of factors such as prior dengue infection, partial vaccination schedules and other traveller-specific considerations. Understanding similarities and differences across national guidance may support more consistent and transparent pre-travel counselling, particularly as travellers encounter conflicting online information.^19^ Comparative analysis of guidance may also assist advisory bodies in countries where guidance is under development, by illustrating how shared evidence has been interpreted and applied in different settings and identifying areas for greater alignment and collaboration.^20^ While a previous perspective article summarized publicly available guidance on Qdenga use in travellers,^8^ it did not examine the processes and considerations underpinning recommendation development. Furthermore, several countries have subsequently developed their first national guidance, while others have updated existing guidance based on evolving evidence and clinical experience.

To better understand variation in travel vaccine recommendations across countries, the International Society of Travel Medicine established a vaccine task force comprising experts with experience in national immunization advisory groups, travel health advisory bodies and travel medicine policy. Following its formation, the task force identified Qdenga as an initial case study because recommendations were emerging internationally and appeared to vary across jurisdictions. This study aimed to compare national guidance on the use of Qdenga for international travellers.

## Methods

A survey-based descriptive approach was used to compare national recommendations for Qdenga in international travellers. Countries were selected based on a combination of high outbound travel volume to dengue-endemic regions and the presence of an established national advisory body responsible for travel vaccine recommendations, including National Immunisation Technical Advisory Groups (NITAGs) or equivalent expert advisory bodies identified through investigator networks. In most cases, a single advisory body per country was included. However, in Germany, guidance from both the NITAG (STIKO, developed in collaboration with the German Society for Tropical Medicine, Travel Medicine and Global Health [DTG] through a shared working group) and the German Society for Travel Medicine (DFR), which issues independent recommendations, was included. Although most included countries represent non-endemic settings, guidance from Thailand and Argentina was also included because of their relevance to international travel medicine: Thailand is a major regional travel hub, and Argentina includes areas with endemic or seasonal dengue transmission alongside significant outbound and regional travel. As the unit of analysis was national/jurisdiction-specific guidance, international recommendations such as World Health Organization (WHO) position papers were not included as separate comparators.

The primary data source was a structured questionnaire (Supplementary Appendix 1  Supplementary  data  are  available  at  JTM  online) capturing key aspects of dengue vaccine guidance relevant to travellers. The questionnaire and analytical framework were developed collaboratively by task force members through virtual meetings and shared online documents. The questionnaire was distributed by email between March and April 2026 to identified members or representatives of relevant advisory bodies. Data collected included regulatory status of Qdenga (e.g. licensed, under review, not approved), characteristics of the advisory body (e.g. remit, composition, decision-making processes), current recommendations for dengue vaccination in travellers, methods used to develop recommendations (e.g. formal systematic review, GRADE methodology, expert consensus), wording of recommendations and links to publicly available guidance documents. Additional information on vaccine access and approximate pricing was also collected where available. Participation was voluntary, and no personal or identifying information about individual respondents was collected.

To enable standardized comparison, respondents were asked to indicate the category that best reflected their advisory body’s recommendation for each of the following traveller groups:

(1) Laboratory-confirmed prior dengue;
(2) Clinical or epidemiological history compatible with prior dengue infection;
(3) No known prior dengue infection (serostatus unknown); and
(4) Laboratory-confirmed seronegative status

For each group, respondents selected from predefined categories (recommended, can be considered, not recommended or not addressed) and provided the exact wording from their national guidance to support their selection.

Publicly available national guidance documents were reviewed, including URLs provided by respondents and, where necessary, additional materials identified through targeted searches of official government, public health and advisory body websites. These documents were used to confirm reported recommendations, clarify conditional or stratified advice and extract additional contextual detail.

Each completed questionnaire was extracted into a predefined Excel template, with one row per advisory body. Initial extraction from completed forms into the template was supported using artificial intelligence (AI)-assisted tools (Microsoft Copilot) to improve efficiency; all entries were subsequently reviewed and manually verified by S.L.M. against the original survey responses and source guidance documents. Where discrepancies or ambiguities were identified, the original guidance text was prioritized.

Data were synthesized into comparative summary tables of national recommendations, with hyperlinks to source documents where applicable. Where recommendations were conditional or stratified—e.g. by duration of travel, time since prior dengue infection or time available before departure—the dominant recommendation was used for tabulation, with important nuances retained in table footnotes or narrative synthesis. Data were analysed descriptively to identify common themes, differences in recommendations and areas of uncertainty across countries and advisory bodies.

## Results

Survey responses were received from 18 advisory bodies across 17 countries between 9 March and 19 May 2026. Guidance on Qdenga was available from 14 countries (12 non-endemic and 2 endemic). Thirteen countries had licensed Qdenga and issued guidance (Table 1), while one (Canada) had issued guidance in the absence of licensure. In Australia, Qdenga had been licensed but guidance was still in development, and Japan and the USA had neither licensure nor national guidance. Germany was represented by two advisory bodies that had issued different guidance. Across countries where Qdenga was licensed, the approved indication was for prevention of dengue disease in individuals aged four years and over. Guidance-issuing bodies included NITAGs, public health institutes, professional societies and travel health advisory bodies.

**Table 1:** Qdenga (TAK-003) licensing and access by country.

Country	Licensed	Licensing authority	License date	Guidance issued	Issuing body	Body type	Most recent guidance date	Guidance URL	Context type ^a^
Australia	Yes	TGA	08-04-2026	In progress	ATAGI	NITAG	N/A	N/A	C
Belgium	Yes	EMA	05-12-2022	Yes	SHC	NITAG	20-Jun-2024	https://www.hgr-css.be/en/report/9739/vaccination-against-dengue	A
Canada	No	N/A	N/A	Yes	CATMAT	Travel Health Advisory Body	14-May-2025	https://www.canada.ca/en/public-health/services/catmat/recommendations-qdenga-dengue-vaccine-jurisdictions-authorized-travellers.html	B
Denmark	Yes	DMA	01-03-2023	Yes	SSI	Public Health Institute	26-Feb-2025	https://www.ssi.dk/aktuelt/nyhedsbreve/epi-nyt/2025/uge-7-9---2025	A
France	Yes	EMA	05-12-2022	Yes	HCSP	NITAG	Apr-2025	https://www.hcsp.fr/explore.cgi/avisrapportsdomaine?clefr=1439	A
Germany^b^	Yes	EMA	5-12-2022	Yes	STIKO-DTG	NITAG	30-Dec-2023	https://www.rki.de/DE/Aktuelles/Publikationen/Epidemiologisches-Bulletin/2023/48_23.html;	A
Germany^b^	Yes	EMA	05-12-2022	Yes	DFR	Professional Society	16-03-2026	https://www.fachgesellschaft-reisemedizin.de/	A
Italy	Yes	EMA/AIFA	23-02-2023	Yes	SIMVIM	Professional Society	16-Jan-2026	https://simvim.org/media/pages/news/aggiornate-indicazioni-utilizzo-vaccino-contro-dengue/abcee2a929-1769986140/indicazioni-utilizzo-vaccino-contro-dengue_rev-16-01-2026.pdf	A
Japan	No	N/A	N/A	No	N/A	N/A	N/A	N/A	D
Netherlands	Yes	EMA	05-12-2022	Yes	LCR	Travel Health Advisory Body	Jan-2025	https://produlcrproduasset.blob.core.windows.net/lcrproductionblob/2025-02/D37%20-%20Dengue%20januari%202025.pdf	A
Portugal	Yes	EMA	05-12-2022	Yes	SPMV	Travel Health Advisory Body	Mar-2026	https://spmv.simposium.pt/recomenda%C3%A7%C3%B5es/	A
Spain	Yes	EMA	05-12-2022	Yes	Ministry of Health and SEIMC-GEPI	Health Ministry/ Professional Society	11-Sep-2024	https://www.sanidad.gob.es/areas/sanidadExterior/laSaludTambienViaja/notasInformativas/docs/NI_ReemergenciaDENGUE-NuevaVacunaFrenteAlDengue-11Septiembre24.pdf; https://seimc.org/wp-content/uploads/2025/05/gepi-dc-2023-Vacunacion_dengue.pdf	A
Sweden	Yes	EMA	05-12-2022	Yes	VEG, Swedish Society for Infectious Diseases Physicians	Professional Society	26-Jan-2026	https://resemedicin.se/wp-content/uploads/2026/03/260126-Vagledning-for-vaccination-med-denguevaccinet-Qdenga.pdf	A
Switzerland	Yes	Swissmedic	29-07-2024	Yes	ECTM	Travel Health Advisory Body	19-Sep-2024	https://smw.ch/index.php/smw/article/view/3858	A
United Kingdom	Yes	MHRA	26-01-2023	Yes	JCVI	NITAG	14-May-2025	https://travelhealthpro.org.uk/media_lib/mlib-uploads/full/the-green-book-chapter-15a-dengue-october-2024.pdf	A
United States	No	N/A	N/A	No	-	N/A	-	-	D
Examples from dengue-endemic countries									
Argentina	Yes	ANMAT	26-04-2023	Yes	CONAIN	NITAG	Sep-2024	https://www.argentina.gob.ar/sites/default/files/09-2024-lt-vacuna-tetravalente-de-dengue.pdf	A
Thailand	Yes	Thai FDA	08-05-2023	Yes	IDAT	Professional Society	12-Mar-2025	https://idthai.org/Contents/Views/?d=!17!9!!1086!	A

^a^Context type: A = Licensed and guidance available; B = Not licensed but guidance available; C = Licensed with guidance in development; D = Not licensed and guidance not available.

^b^Separate rows reflect distinct recommendations from the NITAG (STIKO) and a professional society (DFR). STIKO produces is advised by another professional society (DTG) through a joint working group.

Abbreviations: AIFA, Agenzia Italiana del Farmaco (Italian Medicines Agency); ATAGI, Australian Technical Advisory Group on Immunisation; CATMAT, Canadian Committee to Advise on Tropical Medicine and Travel (CATMAT); CONAIN, Comisión Nacional de Inmunización (National Immunisation Commission); DMA, Danish Medicines Agency; DFR, Deutsche Fachgesellschaft für Reisemedizin (German Society for Travel Medicine); DTG, German Society for Tropical Medicine, Travel Medicine, and Global Health; ECTM, Expert Committee for Travel Medicine; EMA, European Medicines Agency; FDA, Food and Drug Administration; HCSP, Haut Conseil de la Santé Publique (High Council for Public Health); JCVI, Joint Committee on Vaccination and Immunisation; IDAT, Infectious Disease Association of Thailand; LCR, Landelijk Coordinatiepunt Reizigersadvisering (Dutch National Advisory Board on Travel Health); N/A, not applicable; MHRA, Medicines and Healthcare products Regulatory Agency; NITAG, National Immunization Technical Advisory Group; SEIMC-GEPI, Enfermedades Infecciosas y Microbiología Clínica—Grupo de Estudio de Patología Importada (Spanish Society of Infectious Diseases and Clinical Microbiology—Imported Diseases Group); SHC, Superior Health Council; SIMVIM, Società Italiana di Medicina dei Viaggi e delle Migrazioni (Italian Society of Travel and Migration Medicine); SPMV, Sociedade Portuguesa de Medicina do Viajante (Portugese Society of Travel Medicine); SSI, Statens Serum Institut; STIKO, Ständige Impfkommission (Standing Committee on Vaccination); Swissmedic, Swiss Agency for Therapeutic Products; TGA, Therapeutic Goods Administration; Thai FDA, Thai Food and Drug Administration; VEG, Vaccine Expert Group.

### Recommendations by dengue immune status

Advisory bodies differed in how dengue immune status was incorporated into vaccination recommendations for Qdenga (Table 2). Several advisory bodies, including those from Belgium, Canada, Germany-STIKO-DTG, Switzerland and the UK, recommended vaccination for travellers with evidence of prior dengue infection but not those with unknown serostatus or confirmed seronegativity. Advisory bodies from France, the Netherlands, Spain and Sweden allowed vaccination to be considered for selected travellers without documented prior dengue infection, based on factors such as duration or frequency of travel, destination-specific transmission risk, outbreak context and the feasibility of completing the two-dose schedule before departure. Guidance from Portugal recommended vaccination irrespective of serostatus for travellers to areas with active outbreaks or staying in risk areas for >4 weeks. Guidance from the German Society for Travel Medicine (DFR) and Italian Society of Travel and Migration Medicine (SIMVIM) recommended vaccination for travellers to risk areas irrespective of serostatus. Guidance from Argentina and Thailand, both countries with endemic dengue transmission, applied a broader, population-based approach with recommendations driven primarily by epidemiological risk rather than individual serostatus.

**Table 2:** Summary of advisory body recommendations for Qdenga (TAK-003) for travellers.

Country	Recommendations by patient group				Testing approach (serology) ^a^	2-dose series completion before travel ^b^	Age bounds	Timing after prior dengue	Estimated cost per dose (USD) ^c^
	Lab-confirmed prior infection	Clinical/ epidemiological history of dengue	Serostatus unknown	Lab-confirmed seronegative					
Belgium	Recommended	Recommended	Not recommended	Not addressed	No	Preferred	6–60 years	≥6 months	<120
Canada	Can be considered	Not recommended	Not recommended	Not recommended	No	Required	NA	NA	NA
Denmark	Recommended	Recommended	Can be considered	Can be considered	No	Not required	≥4 years	NA	>200
France	Recommended	Recommended	Can be considered	Can be considered	No	Preferred	≥6 years	≥6 months	100–200
Germany (STIKO-DTG)^d^	Recommended	Recommended	Not recommended	Not recommended^e^	No^f^	Required	≥4 years	≥6 months	100–150
Germany (DFR)^d^	Recommended	Recommended	Recommended	Recommended	No	Not required	≥4 years	≥2 months	100–150
Italy (SIMVIM)	Recommended	Recommended	Recommended	Recommended	No	Preferred	≥4 years	≥6–12 months	100–200^g^
Netherlands	Recommended	Recommended	Can be considered	Not addressed	No	Preferred	≥4 years	≥12 months	100–200
Portugal	Recommended	Recommended	Recommended^h^	Recommended^h^	No	Not required	≥4 years	NA	100–150
Spain	Recommended	Can be considered	Can be considered	Can be considered	No	Preferred	≥4 years	NA	100–150
Sweden	Recommended	Recommended	Can be considered	Can be considered^i^	No	Preferred	6–60 years	NA	>200
Switzerland	Recommended	Can be considered	Not recommended	Not recommended	Selectively	Preferred	≥6 years	≥6 months	100–200
United Kingdom	Recommended	Can be considered	Not recommended	Not recommended	Selectively	Preferred	≥4 years	≥12 months	100–200
Examples from dengue-endemic countries									
Argentina	Recommended	Recommended	Recommended	Recommended	No	Preferred	≥4 years	3–6 months	<120
Thailand	Can be considered	Can be considered	Can be considered	Can be considered	No	Not specified	4–60 years	≥6 months	<120

^a^Whether dengue serology is used to determine prior infection for vaccine eligibility. Categories: Routinely = recommended in all cases; Selectively = supported in specific circumstances; No = not needed; Not addressed = not referenced in guidance.

^b^Whether the advisory body recommends completing the full two-dose series before travel to a dengue-endemic area.

^c^Costs are approximate and presented as ranges to reflect variation in pricing across providers, settings and over time, including currency exchange fluctuations.

^d^Separate rows reflect distinct recommendations from the National Immunisation Technical Advisory Group (STIKO) and a professional society (DFR).

^e^If, after thorough medical counselling, vaccination is individually considered, seronegative individuals should be advised that current data have not shown protection against DENV-3/4 in dengue-naïve individuals after vaccination and an increased risk of disease enhancement during a subsequent dengue infection cannot be ruled out. A complete vaccination series should be administered before departure.

^f^Retrospective serological testing not recommended; cross-reactive antibodies (e.g. from TBE vaccine) may yield false positive results

^g^In areas with autochthonous cases, publicly funded targeted vaccination may be available

^h^Recommended irrespective of serostatus for travellers to areas with outbreaks or epidemics, or for those staying in risk areas for >4 weeks.

^i^Vaccine may be considered for seronegative individuals with long-duration exposure (≥2–3 months or frequent shorter trips within 1 year); such individuals should be counselled regarding lower efficacy against DENV-3/4 and potential risks.

Abbreviation: NA, not addressed.

### Traveller, trip and exposure-related factors

Advisory bodies also varied in how traveller, trip and exposure-related factors were incorporated into vaccine recommendations (Table 3). Destination-specific dengue risk was considered by all advisory bodies. Travel duration informed recommendations across multiple countries, with thresholds ranging from 14 days to 3 months. Repeated or cumulative exposure was also incorporated by several advisory bodies, particularly when considering vaccination of travellers without documented prior dengue infection. Some guidance additionally considered traveller categories (e.g. expatriates, military personnel), while host factors associated with severe dengue were explicitly incorporated into recommendations in only a few countries. These factors were combined with dengue immune status in different ways, either to further define eligibility among travellers with prior dengue infection or to broaden eligibility to selected travellers without documented prior infection. Together, they often played a major role in determining vaccine eligibility.

**Table 3:** Incorporation of traveller-, trip- and exposure-related factors into national Qdenga recommendations.

Country/advisory body	Role of traveller-, trip- and exposure-related factors in eligibility	Traveller-, trip- and exposure-related factors considered					Summary of eligibility wording from guidance ^d^
		Destination risk ^a^	Travel duration	Repeated/cumulative exposure	Traveller type ^b^	Host factors ^c^	
**Belgium (SHC)**	Used to refine eligibility; prior dengue infection required	Yes	Yes	Yes	Yes	No	Recommended for people aged ≥6 years with prior dengue undertaking stays >4 weeks or frequent travel to dengue-endemic regions.
**Canada (CATMAT)**	Used to inform individualized risk assessment^e^; prior dengue required	Yes	Yes	Yes	No	No	May be considered for travellers aged ≥4 years with laboratory-confirmed prior dengue who are assessed as having relatively higher exposure risk based on an individualized assessment.
**Denmark (SSI)**	May extend eligibility to selected travellers without prior dengue	Yes	Yes	Yes	Yes	No	May be considered for travellers ≥4 years without previous dengue infection undertaking extended stays (typically ≥4 weeks) including work assignments in high-prevalence areas; counselling regarding evidence limitations is recommended.
**France (HCSP)**	May extend eligibility to selected travellers without prior dengue	Yes	Yes	Yes	No	Yes^f^	Recommended for travellers aged ≥6 years with host risk factors for severe dengue^f^ undertaking stays >4 weeks or repeated travel to areas with active outbreaks or incidence >100/100 000 person-years irrespective of prior dengue history.
**Germany (STIKO-DTG)**	Used to refine eligibility; prior dengue infection required^g^	Yes	Yes	No	Yes	No	Recommended for travellers aged ≥4 years with laboratory-confirmed prior dengue travelling to dengue-endemic areas with increased exposure risk (e.g. prolonged stays or outbreaks).
**Germany (DFR)**	Destination risk is primary determinant; prior dengue infection is not required	Yes	No	No	No	No	Recommended for people aged ≥4 years travelling to dengue-endemic or epidemic areas with a risk of exposure, irrespective of prior dengue infection status.
**Italy (SIMVIM)**	Destination risk is primary determinant; prior dengue infection is not required	Yes	No	No	No	No	Particularly recommended for travellers aged ≥4 years to endemic areas or areas with an ongoing epidemic, regardless of length of stay or prior dengue infection status.
**Netherlands (LCR)**	May extend eligibility to selected travellers without prior dengue	Yes	Yes	Yes	Yes	No	May be considered for travellers without previous dengue undertaking cumulative travel of ≥3 months within 1 year to countries with a dengue vaccination indication; shorter stays (≥3 weeks), discussed only at travellers’ request^h^
**Portugal (SPMV)**	May extend eligibility to selected travellers without prior dengue	Yes	Yes	Yes	Yes	Yes^i^	Recommended for travellers to endemic areas with active outbreaks regardless of duration of stay or prior dengue; otherwise recommended for stays > 4 weeks, frequent or long-term travellers (including expatriates, military personnel, immigrants) and travellers with host risk factors for severe dengue^i^.
**Spain (SEIMC-GEPI)**	May extend eligibility to selected travellers without prior dengue	Yes	Yes	No	No	Yes^j^	Seropositive travellers: Strongly recommended if visiting outbreak or endemic areas with host risk factors for severe dengue^j^; recommended if visiting endemic areas for ≥14 days; Seronegative travellers: Recommended for outbreak areas or high-transmission areas with host risk factors for severe dengue; may be considered for prolonged travel in endemic areas.
**Sweden (VEG)**	May extend eligibility to selected travellers without prior dengue	Yes	Yes	Yes	No	No	Recommended for travellers aged 6–60 years with previous dengue travelling to risk areas; may be considered for those without previous dengue undertaking prolonged (≥2–3 months) or repeated travel, particularly during outbreaks; counselling regarding evidence limitations are recommended.
**Switzerland (ECTM)**	Used to refine eligibility; prior dengue required	Yes	No	No	No	No	May be recommended for travellers aged ≥6 years with evidence of previous dengue and anticipated exposure in a region with significant dengue transmission.
**UK (JCVI)**	Used to refine eligibility; prior dengue required	Yes	No	No	Yes	No	May be considered for individuals aged ≥4 years with evidence of previous dengue travelling to dengue-risk or outbreak areas, or with occupational exposure to dengue virus.

^a^Destination risk refers to destination-specific dengue risk indicators incorporated into guidance, including dengue endemicity, transmission intensity, incidence or the presence of an outbreak or epidemic.

^b^Traveller type includes categories such as expatriates, frequent travellers, occupational travellers, military personnel, aid workers, flight crew, migrants and travellers visiting friends and relatives (VFR travellers).

^c^Host factors refer to age, comorbidities or other characteristics associated with increased risk of severe dengue.

^d^Wording has been adapted from source guidance for brevity while retaining the key eligibility criteria and decision considerations. Readers should consult original guidance documents for complete recommendation wording and context.

^e^Additional factors considered include likelihood of mosquito exposure, ability to adhere to bite prevention measures and traveller values and preferences

^f^Host risk factors for severe dengue listed in guidance include sickle cell disease, other hemoglobinopathies, complicated hypertension, diabetes, obesity, renal failure, chronic cardiopulmonary conditions and/or thrombocytopathy.

^g^STIKO does not make a recommendation regarding vaccination of travellers without prior dengue infection because of limited evidence. Vaccination may nevertheless be considered on an individual basis following detailed counselling, provided the two-dose series can be completed before travel.

^h^Guidance emphasizes counselling regarding uncertainty about long-term protection, the potential need for future booster vaccination, limited evidence of protection against DENV-3 and DENV-4 and a theoretical risk of more severe dengue following vaccination in travellers without previous dengue infection.

^i^Host risk factors for severe dengue listed in guidance include obesity, diabetes mellitus, hypertension, cardiovascular disease, asthma and chronic kidney disease.

^j^Host risk factors for severe dengue listed in guidance include extremes of age, diabetes, hypertension and renal insufficiency.

### Operational aspects of guidance

Among 12 non-endemic countries where Qdenga was licensed and guidance published, minimum age recommendations ranged from 4 to 6 years, aligning with regulatory authorization (e.g. European Medicines Agency [EMA]) or WHO Strategic Advisory Group of Experts on Immunization (SAGE) recommendations.^21^ Two advisory bodies specified an upper age limit of 60 years, while most did not state an upper age restriction.

All advisory bodies recognized the licensed two-dose schedule administered 3 months apart. However, guidance differed in whether completion of both doses was required prior to travel. Canada and Germany-STIKO-DTG recommended vaccination only when the full two-dose series could be completed prior to departure. In contrast, French and Dutch guidance permitted administration of a single dose prior to travel in selected circumstances (e.g. imminent travel to areas with active outbreaks or prolonged exposure), with explicit recommendations to complete the series after travel. Guidance from Germany-DFR adopted a less restrictive clinical approach, explicitly stating that completion of both doses prior to travel was not required.

Approaches to assessing prior dengue infection also varied across advisory bodies. Some restricted vaccination to travellers with laboratory-confirmed prior infection, while others considered a compatible clinical and epidemiological history as sufficient for eligibility. Routine screening serology was generally not recommended due to limitations in test performance, including potential for cross-reactivity with other flaviviruses. Only two advisory bodies (Switzerland, UK) incorporated serology selectively, typically in conjunction with specialist input and with caution regarding interpretation; UK guidance included an algorithm to support assessment of prior dengue infection, including when to use serological testing.

For travellers with recent dengue infection, most guidance recommended delaying vaccination for 6–12 months after infection (e.g. the Netherlands, Switzerland, the UK).

Reported costs of Qdenga ranged from ~USD 100 to USD 215 per dose, with substantial variation across countries.

### Risk assessment domains identified across national guidance

Although recommendations were tabulated primarily by dengue immune status for standardized comparison, synthesis of guidance documents identified a recurrent set of considerations used to assess the appropriateness of Qdenga vaccination for individual travellers. These considerations were evident in recommendation wording, conditional eligibility criteria and accompanying explanatory text but were not consistently presented as a formal framework. Table 4 synthesizes the core decision domains underpinning national guidance and illustrates how differences in their relative weighting and operationalization contribute to variation in recommendations.

**Table 4:** Core decision domains and their operationalization in national guidance on TAK-003 use for travellers from non-endemic settings.

Risk assessment domain	Key traveller considerations	Operationalization in national guidance	Role in determining eligibility for vaccination
***Host-related considerations***			
Prior dengue infection	Evidence of prior dengue infection and implications for vaccine risk–benefit	Operationalized as required, preferred or not required depending on perceived vaccine risk–benefit balance.	Primary determinant in most guidance
Evidence availability and certainty for specific populations	Availability and quality of safety and effectiveness data in specific groups	Restrictions or precautionary recommendations where evidence is limited (e.g. seronegative travellers, older adults)	Driver of restriction or precaution
Host susceptibility to severe dengue	Age, comorbidities and other factors associated with more severe outcomes.	Usually considered alongside exposure risk; explicitly incorporated into a minority of guidelines.	Contextual/modifying factor
Contraindications and precautions	Immunocompromise, pregnancy and other live vaccine contraindications	Applied as exclusion criteria irrespective of exposure risk.	Exclusion criterion
***Travel and exposure considerations***			
Epidemiological exposure risk	Destination-specific dengue transmission intensity, endemicity seasonality, outbreak context	Defined using transmission risk indicators, incidence or outbreak activity.	Secondary determinant of eligibility
Trip characteristics	Duration of travel, cumulative exposure, repeat or long-term travel	Used to refine exposure assessment and define higher-risk travel scenarios.	Contextual/modifying factor
***Vaccine and system considerations***			
Vaccine feasibility and timing	Ability to initiate and complete two-dose schedule before departure	Guidance varies regarding whether completion of primary series before travel is required; some allow single-dose initiation with counselling on partial protection and post-travel completion	Operational constraint influencing eligibility

## Discussion

This study presents a comparative analysis of guidance on the use of the dengue vaccine Qdenga for travellers across multiple countries. By incorporating direct input from representatives of advisory bodies, this study provides insight into the considerations underpinning recommendation development, complementing previous summaries^8^ of publicly available guidance. Across non-endemic settings, guidance was generally more restrictive than regulatory authorization, although some advisory bodies adopted less restrictive approaches. While there was broad agreement in recommending Qdenga for travellers with a history of laboratory-confirmed prior dengue infection, substantial variability was observed in recommendations for travellers without documented prior infection. Although dengue immune status formed the primary axis for most recommendations, eligibility was frequently shaped by its interaction with exposure risk, traveller and trip characteristics and practical considerations such as the feasibility of completing the two-dose schedule before travel.

In Germany, differences between the joint STIKO-DTG and DFR guidance may reflect distinct development processes and differences in the timing of guideline publication. More permissive recommendations (e.g. Germany-DFR, Italy) were among the most recently published and incorporated emerging post-marketing experience with the vaccine, including the absence to date of an identified safety signal suggesting disease enhancement in seronegative individuals. In countries with endemic transmission, recommendations were more strongly driven by epidemiological risk, with less emphasis on individual serostatus.

An important contextual factor underpinning cautious guidance in non-endemic settings is the mismatch between populations in which Qdenga efficacy has been demonstrated and those for whom travel recommendations are being formulated. Phase 3 trials were conducted predominantly among children and adolescents living in dengue-endemic areas, whereas travel guidance targets largely immunologically naïve travellers with heterogenous exposure patterns and a wider age distribution.^14^^,^^16^ In non-endemic adult populations, available data are limited to immunogenicity and safety rather than direct measures of protection against clinical dengue.^22–24^ As a result, recommendations for travellers rely on extrapolation from studies conducted in different populations, introducing uncertainty into risk–benefit assessments and likely contributing to the more restrictive and stratified recommendations observed.

Across guidance from non-endemic countries, prior dengue infection emerged as the dominant determinant of vaccine eligibility. Broad agreement in recommending vaccination for travellers with documented prior infection reflects more favourable vaccine performance in baseline-seropositive individuals.^6^ In contrast, recommendations for travellers without documented infection varied substantially. This caution reflects, in part, experience with the earlier dengue vaccine CYD-TDV (Dengvaxia), for which re-analysis of clinical trial data and subsequent policy guidance identified an increased risk of severe dengue in baseline-seronegative recipients.^10^ Although no such signal has been identified for Qdenga, historical experience with Dengvaxia, together with evidence of lower or uncertain efficacy against DENV-3 and DENV-4 in baseline-seronegative individuals with Qdenga,^17^ appears to have led to a more cautious approach to vaccinating seronegative travellers.^13^

While many advisory bodies chose to incorporate prior dengue infection into their recommendations, approaches to establishing prior infection and the role of serological testing varied. Confirmation of prior dengue infection is often challenging in travellers, as infection can only be reliably established if laboratory testing was performed at the time of illness.^13^ In practice, even travellers with a clinically and epidemiologically compatible history may not have been tested or have documentation of results, and a substantial proportion of infections are asymptomatic.^18^ Retrospective serological testing is limited by cross-reactivity with other flaviviruses and should therefore be interpreted with caution.^25^ Accordingly, most advisory bodies discouraged routine serology, while those incorporating testing did so selectively, e.g. in travellers with a plausible history or increased likelihood of prior exposure, with results interpreted alongside clinical and epidemiological factors. In this context, variation in guidance reflects differing thresholds for accepting probable prior dengue infection in the absence of definitive laboratory confirmation, in addition to uncertainty around the reliability of available serological assays.

Several advisory bodies also addressed the timing of vaccination following recent dengue infection, with recommended delays of 6–12 months reflecting how immune status evolves after primary infection. Primary infection confers lifelong protection against the infecting serotype and a period of short-term cross-protection to other dengue serotypes (heterotypic immunity).^26^ Analyses of longitudinal cohort data from endemic populations suggest that the probability of clinically apparent infection is reduced in the first year following primary infection compared with later periods.^27^ As this protection wanes, subsequent infections become more clinically apparent and are more strongly associated with severe disease. In this context, delaying vaccination can be understood as optimizing vaccine benefit by avoiding immunization during a period of reduced clinical risk.

Age-based recommendations in national guidance were generally aligned with regulatory authorization or WHO SAGE guidance, although approaches differed in how evidence gaps in older adults were addressed. Some advisory bodies specified an upper age threshold or advised against vaccination in travellers over 60 years of age without evidence of prior dengue infection, reflecting limited safety and efficacy data in this population.^21^ Conversely, the lack of an upper age limit in most guidance suggests recognition of the increased risk of severe dengue outcomes with increasing age.^28^^,^^29^

Although all advisory bodies recognized Qdenga as a two-dose schedule administered 3 months apart, approaches differed markedly in whether completion of two doses was required prior to travel. Some guidance restricted vaccination to travellers able to complete both doses before departure, while others allowed a single pre-travel dose in higher-risk scenarios, with explicit counselling regarding partial protection and the importance of post-travel completion. These differences have direct implications for clinical practice, particularly when vaccination is initiated in one setting and completed in another. For example, a traveller may not be eligible to receive a second dose in a different jurisdiction due to differences in age eligibility or requirements for documenting prior dengue infection. Such scenarios highlight how variation in guidance can affect continuity of care and complicate clinical decision-making in cross-border contexts. Emerging post-licensure experience has reported breakthrough dengue infections in travellers following vaccination with one or two doses, reinforcing the importance of counselling travellers that vaccination reduces, but does not eliminate, the risk of dengue and that mosquito avoidance measures remain important regardless of vaccination status.^30^^,^^31^

Overall, advisory bodies addressed similar decision domains—including serostatus-dependent vaccine performance, epidemiological exposure risk, evidence limitations, age-related uncertainty and feasibility of completing vaccination—but weighted and combined them differently. This variation is consistent with broader evidence that immunization advisory bodies draw on similar core criteria but differ in how these are prioritized and operationalized within their decision-making frameworks.^32^ Similar variability has been observed in other areas of travel medicine, where recommendations diverge in response to differing interpretations of evidence, data availability and risk tolerance across advisory groups.^19^^,^^20^ Differences may also reflect variation in recommendation development processes. Some advisory bodies operated within formal national immunization advisory structures using established evidence assessment and evidence-to-decision frameworks, whereas others relied more heavily on expert consensus and professional judgement. Such differences may influence how uncertainty is interpreted and translated into recommendations, particularly when evidence is limited or indirect. Variation may also reflect regulatory processes, advisory structures and system-level factors such as access to diagnostic testing and medicolegal or policy contexts in which guidance is developed.

Travellers may encounter conflicting recommendations online, and clinicians may be required to interpret and reconcile this guidance when advising across different care contexts. Variation in recommendations based on similar underlying evidence may also influence perceptions of consistency and trustworthiness of guidance in an evolving and increasingly visible global advisory landscape.^33^^,^^34^ While some advisory bodies published detailed evidence-to-recommendation summaries through formal processes, others provided limited publicly available justification, reducing transparency around how trade-offs were resolved. Greater transparency regarding how evidence and judgement shape recommendations may support clearer counselling, facilitate shared decision-making between clinicians and travellers and may also support greater alignment and collaboration across advisory bodies over time.^13^

This study has some limitations. Guidance continues to evolve, and recommendations were assessed at a single point in time. In addition, variability in the level of publicly available documentation means that some decision-making processes could only be inferred from recommendation wording rather than fully reconstructed. Although triangulation of survey responses with source documents enabled systematic comparison across jurisdictions, some contextual factors may not have been fully captured. Finally, certain countries (e.g. France) include regions or territories with endemic dengue transmission; however, guidance specific to populations in these settings was not examined separately in this analysis.

## Conclusion

Currently, Qdenga is the only dengue vaccine with indications extending to travellers, and important knowledge gaps remain. Further data on dengue infection rates and clinical outcomes among baseline-seronegative travellers receiving one or two doses will be particularly informative, as will evidence on protection against all four virus serotypes and outcomes in older adults. Post-licensure surveillance, registry-based studies of imported infections and data from travel surveillance networks such as GeoSentinel may contribute critical traveller-specific evidence. In parallel, emerging data from other dengue vaccines may further inform future recommendations. As the evidence base evolves, guidance is likely to be refined. Until then, transparency in how evidence limitations and practical trade-offs are translated into recommendations may be as important as the recommendations themselves.

## Supplementary Material

Supplementary_materials_taag065

## Data Availability

Data underlying this study are derived from survey responses and publicly available national guidance documents. Extracted data supporting the findings are available within the manuscript. The survey instrument is provided in the Supplementary Appendix.
