# Supplementary material for: Shared evidence, different recommendations: a comparative analysis of Qdenga (TAK-003) dengue vaccine guidance for travellers across countries
Source: J Travel Med. 2026 Jul 22;33(6):taag065. doi: 10.1093/jtm/taag065 (PMC13621450; doi:10.1093/jtm/taag065)
Supplement: Supplementary file 1 [file supplementary_materials_taag065.docx]

**Dengue Vaccine (Qdenga) Survey**

*Version 4.0 | 2026-03-02*

**Purpose:** To collect standardised, comparable information on national guidance for the Qdenga dengue vaccine from NITAGs and other expert advisory groups.

**Instructions:**

- Please complete one form per advisory body issuing recommendations.
- Use the check boxes (☐) and short text fields provided. For URLs, include a public link wherever possible.
- Some fields may be pre‑filled 🡪 please correct any errors and update missing items.

**Section A – Country details & Qdenga licensing status**

| **ITEM** | **RESPONSE** |
| --- | --- |
| **Country/territory** |  |
| **Is Qdenga (TAK-003) licensed in your country?** (please check one) | ☐ Yes 🡪 *please continue*  ☐ No 🡪 *skip to section B*  ☐ Under review / in progress 🡪 *skip to section B* |
| **If YES – Name of licensing authority** |  |
| **Date of licensing (DD-MM-YYYY)** |  |
| **Licensed indications(s)** |  |
| **URL for licensing details** |  |
| **Is Qdenga currently available on the market?** (please check one) | ☐ Yes ☐ No  ☐ Limited availability (please specify): |

**Section B – Advisory body and recommendations in your country**

| **ITEM** | **RESPONSE** |
| --- | --- |
| **Name of advisory body that you represent:** |  |
| **Has your advisory body issued guidance for Qdenga?** (please check one): | ☐ Yes 🡪 *please continue*  ☐ No 🡪 *skip to Section E* |
| **Level of this body** (please check one): | ☐ National  ☐ Sub-national (please specify jurisdiction: _____) |
| **Type of body** (please check one): | ☐ NITAG  ☐ Ministry of Health  ☐ Professional Society  ☐ Public Health Institute  ☐ Other, please specify: ________________ |
| **Date of most recent guidance** (DD-MM-YYYY): |  |
| **URL for most recent guidance** (web link): |  |
| **Are there other bodies in your country that issues vaccine recommendations?** | ☐ Yes ☐ No  If YES, please list name(s):  If this body has issued guidance on Qdenga, please provide a URL and/or suggested contact person: |

**Section C – Recommendations for use of Qdenga**

| **ITEM** | | **RESPONSE** | | | |
| --- | --- | --- | --- | --- | --- |
| **Summary of recommendation wording (target population)** | | *Please copy/paste the exact wording from your national recommendation (if available).* | | | |
| **For each of the following groups, please check the box that best reflects the recommendation issued by your advisory body:** | | | | | |
| **Group** | **Recommended** | | **Can be considered** | **Not recommended** | **Not addressed** |
| Laboratory-confirmed prior dengue infection | ☐ | | ☐ | ☐ | ☐ |
| Clinical/epidemiological history compatible with prior dengue infection | ☐ | | ☐ | ☐ | ☐ |
| No known prior dengue infection (serostatus unknown) | ☐ | | ☐ | ☐ | ☐ |
| Laboratory-confirmed seronegative | ☐ | | ☐ | ☐ | ☐ |
| **Lower age limit** | |  | | | |
| **Upper age limit** | | *Insert “none” if no upper limit* | | | |
| **Is testing for prior dengue infection recommended? If yes, what method(s)?** | |  | | | |
| **Recommended schedule** | | *Short text (e.g. 2 doses, 3 months apart)* | | | |
| **Is completion of the 2-dose series before travel required?** (please check one) | | ☐ Yes ☐ Preferred  ☐ Not required ☐ Not specified | | | |
| **Minimum interval between final dose and travel (if any)** | |  | | | |
| **Recommendations on timing after prior dengue infection (if any)** | | *E.g., minimum interval before vaccination, if specified* | | | |
| **Listed contraindications**  (please check all that apply) | | Pregnancy: ☐ Yes ☐ No  Breastfeeding: ☐ Yes ☐ No  Immunocompromise: ☐ Yes ☐ No  Children under 4 years: ☐ Yes ☐ No  Other: ________ | | | |
| **Advice on co-administration with other vaccines (if any)** | |  | | | |

**Section D – How Qdenga recommendations were developed**

| **ITEM** | **RESPONSE** |
| --- | --- |
| **How were recommendations developed?** | *E.g. PICO questions, structured evidence reviews, expert consensus* |
| **Summary of evidence base** | *Key data sources, certainty of evidence, major evidence gaps, key judgements* |
| **Are evidence summaries publicly available?** (please check all that apply) | ☐ Yes ☐ No |
| **If yes – URL** |  |
| **Important contextual factors or key judgement areas** |  |
| **How can travellers access Qdenga in your country?** | *E.g. provider types, private/public access, insurance coverage* |
| **Approximate cost per dose in $USD**  **(and factors affecting price if variable)** |  |

**Section E – Where no current guidance exists**

*Please complete this section ONLY if your advisory body is yet to issue Qdenga guidance*

| **ITEM** | **RESPONSE** |
| --- | --- |
| **What is the status of Qdenga guidance for your advisory body?** | ☐ Guidance currently in development  ☐ Guidance planned, but development not yet started  ☐ Unsure / under consideration |
| **Is your advisory body currently following any external guidance for Qdenga?** | ☐ Yes ☐ No |
| **If YES – please indicate which external sources are being referenced:** | ☐ WHO position statement:  ☐ Guidance from another country (please specify): _____  ☐ Other: __________ |
| **Is there any interim advice in place?** | ☐ Yes ☐ No  *If YES – please provide brief details, e.g. URL and summary:* |
